# Small extracellular vesicle-loaded bevacizumab reduces the frequency of intravitreal injection required for diabetic retinopathy

**DOI:** 10.7150/thno.78426

**Published:** 2023-04-09

**Authors:** Shivakumar K Reddy, Abhijna R Ballal, S Shailaja, Raviraja N Seetharam, Chandrashekar H Raghu, Runali Sankhe, Kanthilatha Pai, Tenzin Tender, Mary Mathew, Annayya Aroor, Ashok K Shetty, Shalini Adiga, Vasudha Devi, Manjunatha S Muttigi, Dinesh Upadhya

**Affiliations:** 1 Centre for Molecular Neurosciences, Kasturba Medical College, Manipal Academy of Higher Education, Manipal, 576104, India.; 2 Department of Ophthalmology, Kasturba Medical College, Manipal Academy of Higher Education, Manipal, 576104, India.; 3 Centre for Biotherapeutics Research, Manipal Academy of Higher Education, Manipal, 576104, India.; 4 Department of Pharmaceutical Biotechnology, Manipal College of Pharmaceutical Sciences, Manipal Academy of Higher Education, Manipal, 576104, India.; 5 Department of Pharmacology, Manipal College of Pharmaceutical Sciences, Manipal Academy of Higher Education, Manipal, 576104, India.; 6 Department of Pathology, Kasturba Medical College, Manipal Academy of Higher Education, Manipal, 576104, India.; 7 Divison of Endocrinology, Diabetes and Metabolism, Department of Medicine, School of Medicine, University of Missouri, Columbia, MO 65211, USA.; 8 Institute for Regenerative Medicine, Department of Molecular and Cellular Medicine, College of Medicine, Texas A&M University Health Science Center, College Station, TX, United States.; 9 Department of Pharmacology, Kasturba Medical College, Manipal Academy of Higher Education, Manipal, 576104, India.

**Keywords:** Diabetic retinopathy, extracellular vesicles, bevacizumab, intravitreal injection, neovascularization.

## Abstract

Diabetic retinopathy (DR) is associated with retinal neovascularization, hard exudates, inflammation, oxidative stress and cell death, leading to vision loss. Anti-vascular endothelial growth factor (Anti-VEGF) therapy through repeated intravitreal injections is an established treatment for reducing VEGF levels in the retina for inhibiting neovascularization and leakage of hard exudates to prevent vision loss. Although anti-VEGF therapy has several clinical benefits, its monthly injection potentially causes devastating ocular complications, including trauma, intraocular hemorrhage, retinal detachment, endophthalmitis, etc.

**Methods:** As mesenchymal stem cells (MSCs) and MSC-derived extracellular vesicles (MSC-EVs) demonstrated safety in clinical studies, we have tested the efficacy of MSC-derived small EVs (MSC-sEVs) loaded anti-VEGF drug bevacizumab in a rat model of DR.

**Results:** The study identified a clinically significant finding that sEV loaded with bevacizumab reduces the frequency of intravitreal injection required for treating diabetic retinopathy. The sustained effect is observed from the reduced levels of VEGF, exudates and leukostasis for more than two months following intravitreal injection of sEV loaded with bevacizumab, while bevacizumab alone could maintain reduced levels for about one month. Furthermore, retinal cell death was consistently lower in this period than only bevacizumab.

**Conclusion:** This study provided significant evidence for the prolonged benefits of sEVs as a drug delivery system. Also, EV-mediated drug delivery systems could be considered for clinical application of retinal diseases as they maintain vitreous clarity in the light path due to their composition being similar to cells.

## Introduction

Diabetes mellitus is a significant global health burden. International Diabetes Federation data shows that 537 million adults (20-79 years) will live with diabetes in 2021, which may rise to 643 million by 2030 and 783 million by 2045 1, 2. The increasing incidence of diabetes, prolonged hyperglycemia, hypertension, dyslipidemia, poor glycemic control, late diagnosis, and impairment of daily lifestyle due to diabetes are the potential risk factors for microvascular complications such as diabetic retinopathy, nephropathy, and neuropathy 1-3. In the eye, diabetes affects many components, but primary vision-threatening pathology occurs in the retina 4.

Diabetic Retinopathy (DR) is one of the most common microvascular complications of both types of diabetes. It remains a leading cause of severe vision loss and blindness in working-age people. DR leads to vision loss through abnormal new blood vessel formation through neovascularization in the retina, exudate formation and associated inflammation, oxidative stress and enhanced retinal cell death 5-8. Anti-VEGF therapy through intravitreal injections is an established treatment for reducing VEGF levels in the retina to prevent neovascularization and hard exudates in diabetic macular edema associated with vision loss in DR 9-14. Although clinically anti-VEGF therapy has several advantages, it is initially required monthly for controlling VEGF levels 15-17. Repeated intravitreal injections potentially cause devastating ocular complications, including trauma, intraocular haemorrhage, retinal detachment, and endophthalmitis 18-21. Thus, there is an essential need for a drug delivery system that could cross the blood-retinal barrier and sustain drug availability for a longer duration to reduce the frequency of intravitreal injections 15, 22-25. Several novel drug delivery strategies are currently adopted to reduce the burden of monthly intravitreal injections 15, 24-25.

Extracellular vesicles are considered next-generation drug delivery platforms 26-27. Mesenchymal stem cell-derived small extracellular vesicles (MSC - sEVs) with a size below 200 nm possess tremendous potential to carry drugs for treating many diseases. This ability is due to their biocompatibility, inherent immunomodulatory functions, and low immuno-stimulatory surface markers 28-29. Previous studies have shown that intravitreally injected MSC-EVs reached different retinal layers, and their presence was detected for several weeks 30-32. This suggests the possibility of MSC-EVs as one of the finest delivery systems to carry drugs for treating diabetic retinopathy. In the present study, we have loaded therapeutic bevacizumab into human bone marrow-derived MSC-sEVs and evaluated their therapeutic efficacy in reducing the frequency of intravitreal injections in a streptozotocin-induced model of diabetic retinopathy in rats.

## Materials and methods

**Figure 1:**
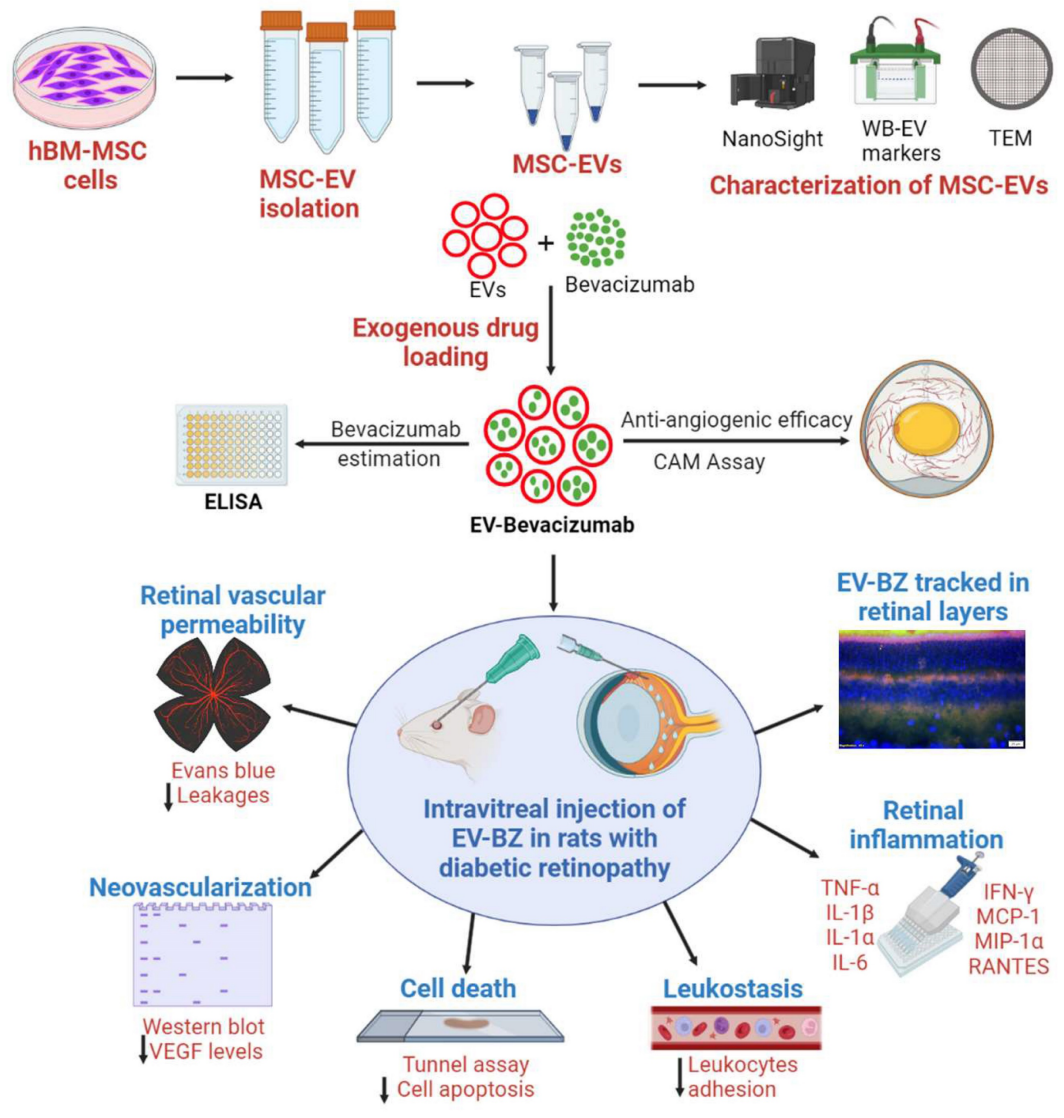

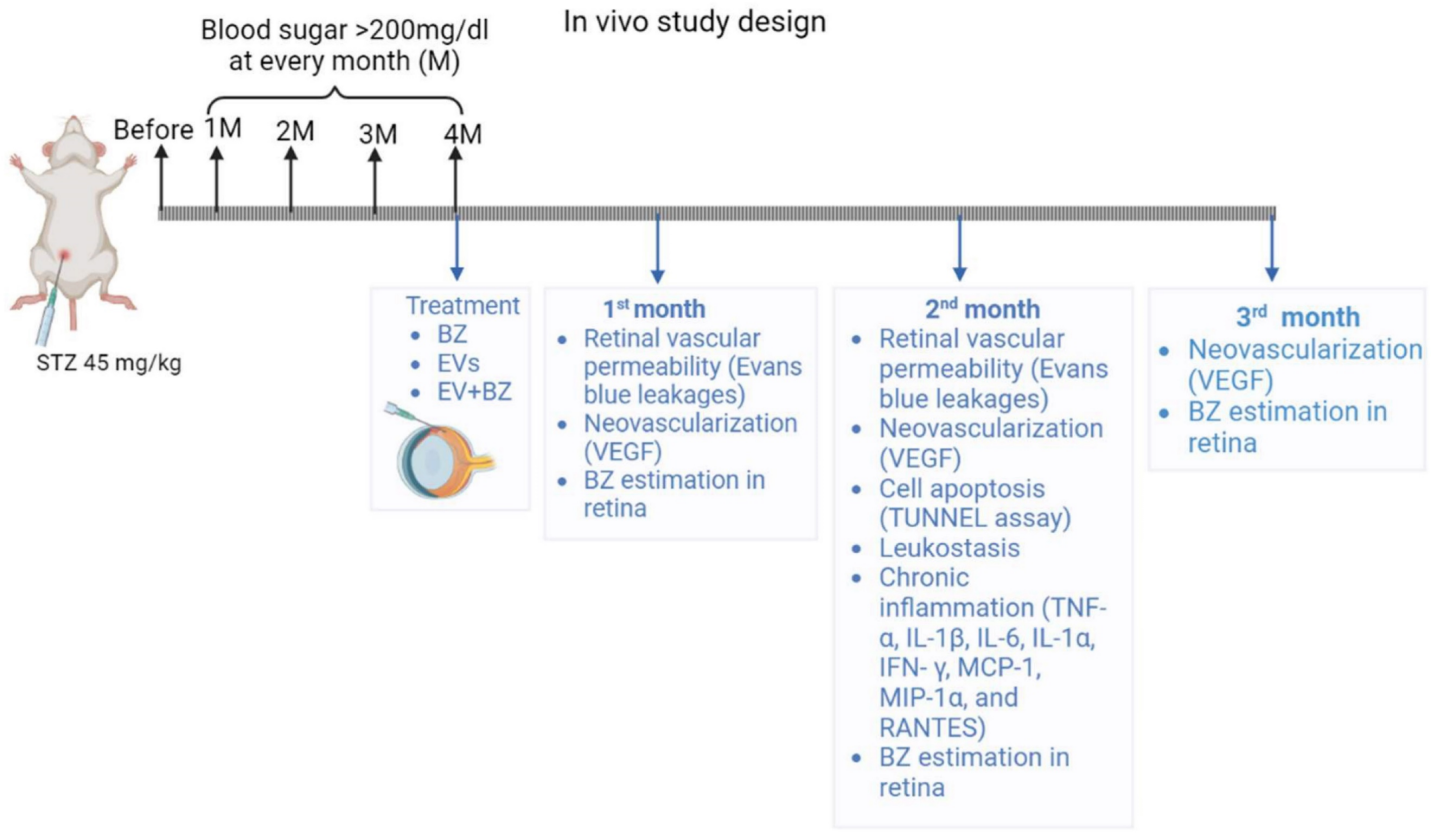
** Experimental outline for *in vitro* and *in vivo* studies.** Human bone marrow derived MSCs were cultured (n = 14 experiments), EVs were isolated and characterized for size, molecular markers, and morphology. The selected sEVs were exogenously loaded with bevacizumab (BZ) using different methods. EV-BZ was tested for efficacy using CAM assay. For tracking EV-BZ in the retina, BZ was conjugated with FITC, and EVs were marked with PKH 26. The therapeutic efficacy of EV-BZ was evaluated in streptozotocin induced rat model of diabetic retinopathy (DR). Anti-angiogenic efficacy was tested using VEGF estimation in the retinal lysates (DR, DR+EVs, DR+ BZ, and DR+EV-BZ groups) at 1, 2 and 3 months following intravitreal administration. The number of retinal leakage sites was evaluated using Evans blue tracer method using retinal whole mounts (in control, DR, DR+BZ, MSC-EVs and DR+EV-BZ groups) at 1 and 2 months following intravitreal administration. Retinal leukostasis was evaluated by perfusion with concanavalin A in control, DR, DR+ BZ, DR+EVs, and DR+EV-BZ groups at one and two months. Retinal cell death was evaluated using TUNEL assay (in control, DR, DR+ BZ, DR+EVs, and DR+EV-BZ groups) at 2 months. The anti-inflammatory potential of EV-BZ was evaluated using rat inflammation ELISA strips (in control, DR, DR+ BZ, DR+EVs, and DR+EV-BZ groups) at 2 months. Specific *in vivo* studies and timelines were provided in Supplementary Figure 1.

A detailed experimental plan with MSC-EVs and EV-loaded bevacizumab (EV - BZ) is provided in Figure 1. The study design and timeline for the *in vivo* work are provided in the Figure S1.

### Culturing human bone marrow derived MSCs for extracellular vesicles

A frozen vial of passage number four hMSCs was thawed at 37 °C, and 2 million cells were seeded in 152 cm^2^ diameter culture dish plates (cat. # 430599; Corning, NY, USA) with complete culture medium (CCM). The CCM medium consists of α-minimal essential media (α - MEM; Gibco), 10% fetal bovine serum (FBS; Gibco), and 1% of 2 mM L - glutamine (Gibco) and Pen Strep (Gibco). Media was replaced on alternative days until cells attained ~70% confluency. At ~70% confluency, media was replaced with protein-free, serum-free Chinese hamster ovary cells media (CD - CHO Medium, cat. # 10743-002; Invitrogen). The conditioned medium was recovered after 6 h and discarded. The media was replaced with fresh CD - CHO media and recovered at two-time points in 48 h. The collected media is processed for further experiment or stored at - 80 °C for further use.

### Isolation of extracellular vesicles

Extracellular vesicles were isolated using the established method 33 - 37. Briefly, the collected fresh media (50 mL) was centrifuged at 2500 x g for 30 min to remove cell debris and larger vesicles. An equal amount of 2 x of 12% PEG (w / v, 6000, Sigma-Aldrich 81260) in 1M NaCl was added to the media, mixed thoroughly, and kept for 14 h at 4 °C. After overnight incubation, the solution was centrifuged in the tabletop centrifuge at 3300 x g at 4 °C for one hour. The supernatant was discarded. The pellet was suspended with 3 mL of DPBS and an equal amount of 2 x of 5% PEG, incubated for an hour at 4 °C and centrifuged again at a speed of 3300 x g at 4 °C for 1h. Following centrifugation, the supernatant was discarded, and the pellet was suspended in 200 µL of PBS (PH-7.2) and used for various experiments.

### Characterization of extracellular vesicles

Isolated EVs were characterized as per MISEV 2018 guidelines 38. The average size and concentration of isolated EVs were determined by nanoparticle tracking analysis using NanoSight LM10 (Malvern Instruments, UK). Samples were vortexed to avoid clump and diluted up to 1 : 10000 with sterile DPBS to achieve consistent particle concentration within the optimal detection range. Fourteen experiments were performed to identify and confirm the number and size of extracellular vesicles isolated for MSCs using this method.

### Morphology of EVs

The ultrastructure of the isolated EVs was evaluated using transmission electron microscopy. Briefly, the sEVs sample was diluted in a 1 : 5 ratio (approximately 1.5 x 10^11^ sEVs), and a few drops of EV suspension were applied to 300 mesh carbon-coated grids at room temperature. After five minutes, excess fluid was removed by blotting with filter paper and washed twice with distilled water. The carbon-coated grids were stained with continuous dripping of 0.5% uranyl acetate, and the excess solution was blotted, and air dried for 10 min at room temperature. The images were collected using Make Jeol Model JM 2100, a multipurpose 120 KV analytical transmission microscope.

### Evaluation of extracellular vesicle-specific marker expression in the isolated sEVs

The expression of positive markers for sEVs, such as TSG 101, CD 63 proteins, and negative marker GRP 94, was confirmed with western blotting. Briefly, the EV and cell samples were lysed with RIPA and proteinase cocktail (Sigma-Aldrich) lysis buffer, sonicated, and proteins were separated based on size through SDS-PAGE electrophoresis and transferred protein to the PVDF membrane. Further, the membranes were probed with rabbit polyclonal antibodies CD 63 (Catalogue no SAB 4301607, Invitrogen), TSG 101 (Catalogue no, PA 531260, Invitrogen), and GRP 94 (Catalogue no, SAB 2101094, Invitrogen) and with appropriate secondary antibodies. The blots confirmed the housekeeping protein expression with mouse monoclonal β-actin (Catalogue no MA 1140, Invitrogen).

### Drug loading to Extracellular Vesicles

Four different methods were used to load the anti-VEGF drug bevacizumab (BZ) (Roche Ltd, Switzerland) into EVs 39-40 with some modifications. 1) freeze-thaw cycle, 2) incubation at room temperature (RT), 3) incubation with 0.2% saponin, and 4) sonication. In the freeze-thaw cycle, 250 µg / mL BZ was loaded into 50 µg (in 250 µL PBS) of EV protein in a 1 : 1 ratio. This mixture was incubated at RT for 30 min, followed by freezing at - 80 ^°^C for 30 min, and the freeze-thaw cycles were repeated three times. BZ and MSC - sEVs were co-incubated at RT for one hour in the RT co-incubation method. In treatment with the saponin method, BZ was incubated with permeabilizing agent 0.2% saponin on a shaker at RT for 30 min. In the sonication method, BZ and sEVs were sonicated (500 v, 2 kHz, 20% power, 4 cycles, 4 s pulse, and 2 s pause) and cooled down on the ice for 45 min to close formed pores during sonication. Following incubation and washing with 2 x of 5% PEG for I h, the total BZ concentration in EVs was estimated using an ELISA method.

### Chorioallantoic membrane Assay

To check the antiangiogenic efficacy sEVs loaded BZ (EV - BZ), we have performed a chorioallantoic membrane (CAM) assay. For this, sharp fine-toothed forceps were used to make an approximate 0.5 cm^2^ square window on day 9-post fertilization of chicken eggs. An incision was made on the eggshell in a portable biosafety cabinet under sterile conditions. VEGF (50 ng) was used as a positive control, VEGF - BZ (50 ng VEGF + 10 µL BZ from 200 mg / mL), MSC - EVs (Equivalent to 50 µg of EV protein), and VEGF - EV - BZ (50 ng VEGF + 10 µL of EV - BZ, equivalent to 50 µg of EV protein containing 60 - 74 µg / mL of BZ) were packed in a 2% agarose disk. Angiogenesis was observed by monitoring blood vessel density, including several branching points from each major vessel in the CAM membrane at 0, 24, and 48 h time points using AngioTool software.

### Conjugation of bevacizumab with FITC

Conjugation of BZ with FITC occurs through the free amino group of the antibody by forming stable thiourea bonds. 250 µL of FITC solution (1mg / mL) in 0.1M bicarbonate/carbonate buffer (pH 9) was mixed with 1 mL of BZ (1 mg/mL) in the same buffer. The solution was incubated at RT on a shaker for 2 h, protected from light. Then, FITC conjugated BZ (BZ - FITC) was purified from free FITC on a size exclusion chromatography through sephacryl 200 HR column (Bio-Rad). One mL of the reaction mixture was applied to the top of the column and was eluted with 10 mM PBS (pH 7.4), 4-5 fractions were collected, and free FITC was eluted only after 70 - 80 mL of PBS washing. The concentration of BZ was determined by measuring the sample absorbance at 280 nm using a standard BSA curve 41.

### Labeling of sEVs

Lipophilic highly fluorescent membrane staining red cell linker dye PKH 26 was used to label sEVs (Sigma -Aldrich, Munich, Germany). Briefly, sEVs (equivalent to 50 µg of EV protein) was diluted in PBS before 1 mL of diluent C was added. In the separate tube, 4 µL of PKH 26 dye was added to 1 mL of diluent C. Both solutions were gently mixed for 5 min, and 5 mL of 1% BSA was added to bind the excessive dye. Solutions were transferred to 300 kDa amicon filters and centrifuged at 4000 x g for 40 min and washed twice with PBS before use.

### Intravitreal administration of small extracellular vesicles loaded with bevacizumab

The BZ-FITC was loaded into PKH 26 labelled sEVs (EV - BZ - FITC) for tracking in the retinal layers. Naïve rats were anesthetized with a cocktail of ketamine (80 mg / kg) and xylazine (8 mg / kg), followed by an intravitreal injection of 10 µL of EV - BZ - FITC (Equivalent to 50 µg of EV protein containing 60 - 74 µg / mL of BZ) with a 32 G syringe (BD Biosciences). Povidone-iodine eye drops were applied to avoid infections after intravitreal injection. At 24 h, eyes were enucleated after intra-cardiac perfusion, and 10 µm retinal sections were taken in a cryostat. Sections were stained with DAPI, and images were taken using a fluorescence microscope.

### Induction of Diabetic retinopathy

Four-month-old albino Wistar rats (200 - 250 g) were used for the entire study. Rats were housed in a cage at standard temperature and humid conditions with 12 h light / dark cycles with chow and water ad libitum. Following a week of acclimatization, diabetes was induced by a single dose of intraperitoneal injection of streptozotocin (STZ, 45 mg / kg). To avoid severe hypoglycemia due to STZ injection, 10% sucrose was supplemented in water, and the blood sugar levels were monitored on the 7^th^ day, 1, 2, 3, and 4 months after STZ injection. The rats showing blood sugar levels of more than 200 mg / dL at all time points were included in the study. We have induced diabetes in 172 rats. Out of 172, 163 rats successfully developed diabetic retinopathy at 4 months following STZ administration, and nine were excluded from the study because of low blood sugar levels. DR - developed rats were randomly categorized into DR groups, one month, two months, and 3 months after intravitreal administration of sEVs, (50 µg equivalent proteins) BZ (10 µL, 200 µg / mL), and EV - BZ (50 µg of sEVs equivalent proteins in 10 µL, containing 60-74 µg / mL of BZ).

### Western blot analysis

Retinal samples were lysed with cock tail of radioimmunoprecipitation assay (RIPA) buffer with protease inhibitor (Sigma, St Louis, MO), and total protein concentration was estimated by a BCA protein assay kit (23227, Pierce Thermo Fisher). An equal amount of protein (15 µg) from each group was separated in sodium dodecyl sulfate-polyacrylamide gel electrophoresis (SDS-PAGE) and transferred to polyvinylidene difluoride membrane (PVDF, Bio-Rad Laboratories). The primary antibody VEGF (MA1-16629, Invitrogen), beta-actin (MA1-140, Invitrogen), and appropriate secondary antibodies were used. The protein was detected with a chemiluminescence reagent (Bio-Rad Laboratories) and hydrogen peroxide. Beta-actin from the same immunoblot was a loading control, and band intensity was analyzed with ImageJ software.

### Evaluation of vascular permeability by Evans blue perfusion

Rats (n = 5 / per group) were euthanized with a cocktail of ketamine (100 mg/kg) and xylazine (8 mg/kg). An incision on the chest was made to expose the heart. Evans blue (Catalogue number; E 2129, Sigma, St Louis, MO) 1 mL from 50 mg/mL was injected into the left ventricle, perfusion for 10 min. The eyeballs were enucleated and fixed in 4% PFA overnight. The next day eyeball was moved to PBS, cornea, and lens were removed by making nicks on the iridocorneal junction. The whole retina was extracted, mounted on the slide, and observed under a fluorescent microscope (Olympus, Tokyo, Japan). The number of capillary leakages was counted in the whole retina in all the groups.

### Evaluation of retinal leukostasis by intracardiac perfusion with concanavalin A

The rats (n = 5) were anesthetized with ketamine (100 mg / kg) and xylazine (8 mg / kg) cocktail, and an incision was made on the abdomen wall to access the chest cavity. Intracardiac perfusion was performed with 50 - 60 mL of pre-warmed saline (0.9% NaCl) for three min to remove the circulating red blood cells and leukocytes. This was followed by perfusion of 10 mL from 100 µg / mL of Concanavalin A Tetramethyl rhodamine conjugate (C 860, Invitrogen) for an additional two minutes. The eyes were enucleated and fixed in the 4% PFA for 2 h. The cornea and lens were separated, and the whole retina was extracted and mounted on the slide. Retinal images were taken using a fluorescent microscope (Olympus, Tokyo, Japan), and the number of adherent leukocytes was counted from the whole retina in each group.

### Estimation of BZ concentration in the retina

One month, two months, and three months after treatment, rats were euthanized, eyeballs were enucleated, and the cornea and lens were separated from the eyeball. In chilled PBS, the whole retina was extracted and lysed in RIPA with protease (Sigma, St Louis, MO) lysis buffer, sonicated, and centrifuged at 10000 rpm for 20 min; the supernatant was collected in the new tube. ELISA method was used to measure the bevacizumab from the retinal lysate. Briefly, VEGF was coated to 96 well plates, kept overnight at 4 ^o^C, and washed wells with PBS three times; the 10 µg (100 µL) samples were added and incubated for 1 h at RT. After three PBS washes, a secondary antibody (1:10000) was added, incubated for 90 min, and washed thrice five min each with PBS. TMB substrate was added to the wells and kept on the shaker for 30 min; once colour developed, the reaction was stopped with a stop solution. The reaction colour changed from blue to yellow, and absorbance was taken at 450 nm.

### Evaluation of retinal cell death

Terminal deoxynucleotidyl transferase (TdT)-mediated dUTP nick end labelling (TUNEL) assay was performed to see cell apoptosis in the retina. *In situ* apoptosis detection kit (MK505, Takara, Japan) was used as per the manufacturer's protocol. Briefly, after deparaffinization (three times in xylene and descending grades of alcohol five minutes each), retinal sections (5 µm) were treated with proteinase K (100 µg/mL) for 15 min at RT and washed with PBS. Sections were incubated with a TUNEL reaction mixture for 90 min at 37 °C, and the reaction was terminated by washing with PBS. Cell nuclei were labelled with DAPI, and TUNEL-positive cells were visualized under a fluorescent microscope with a 40 X objective (Olympus, Tokyo, Japan) and quantified.

### Evaluation of retinal cytokine levels

We have evaluated the cytokines (TNF - α, IL - 1α, IL - 1β, IFN - γ, IL - 6, MCP - 1, MIP - 1α, and rantes) levels in the retina by rat inflammation ELISA strip (EA-1201, Signosis). Following euthanasia, the eyes were enucleated, and the retina was extracted from the eyeball. The retinal sample was sonicated in cold lysis buffer with protease inhibitor (Sigma) and centrifuged at 10000 x g for 5 min at 4 °C. The supernatant was collected, the total protein concentration was measured using a BCA kit, and cell lysate was diluted for the required concentration. The wells were pre-coated with specific cytokine antibodies while each well received 100 µL retinal lysate (10 µg protein). This assay was performed as per the manufacturer's guidelines. In this assay, the cytokine concentration was directly proportional to the intensity of color developed.

For the estimation of TNF - α (EA - 3001; Signosis), and IL1 - β (EA - 3005; Signosis), quantitative ELISA was performed with specific standards and retinal lysates. The assay was performed as per the manufacturer's guidelines. The TNF - α and IL - 1β in the retina were estimated using the standard graph and expressed as pg/mg of protein.

### Statistical analysis

Results were analyzed using GraphPad Prism. All the data were expressed as mean ± standard error of the mean (SEM). One-way analysis of variance and unpaired t-test was used to find the significant difference between the study groups. p < 0.05 was considered statistically significant.

## Results

### Isolated particles from MSCs were small extracellular vesicles

**Figure 2:**
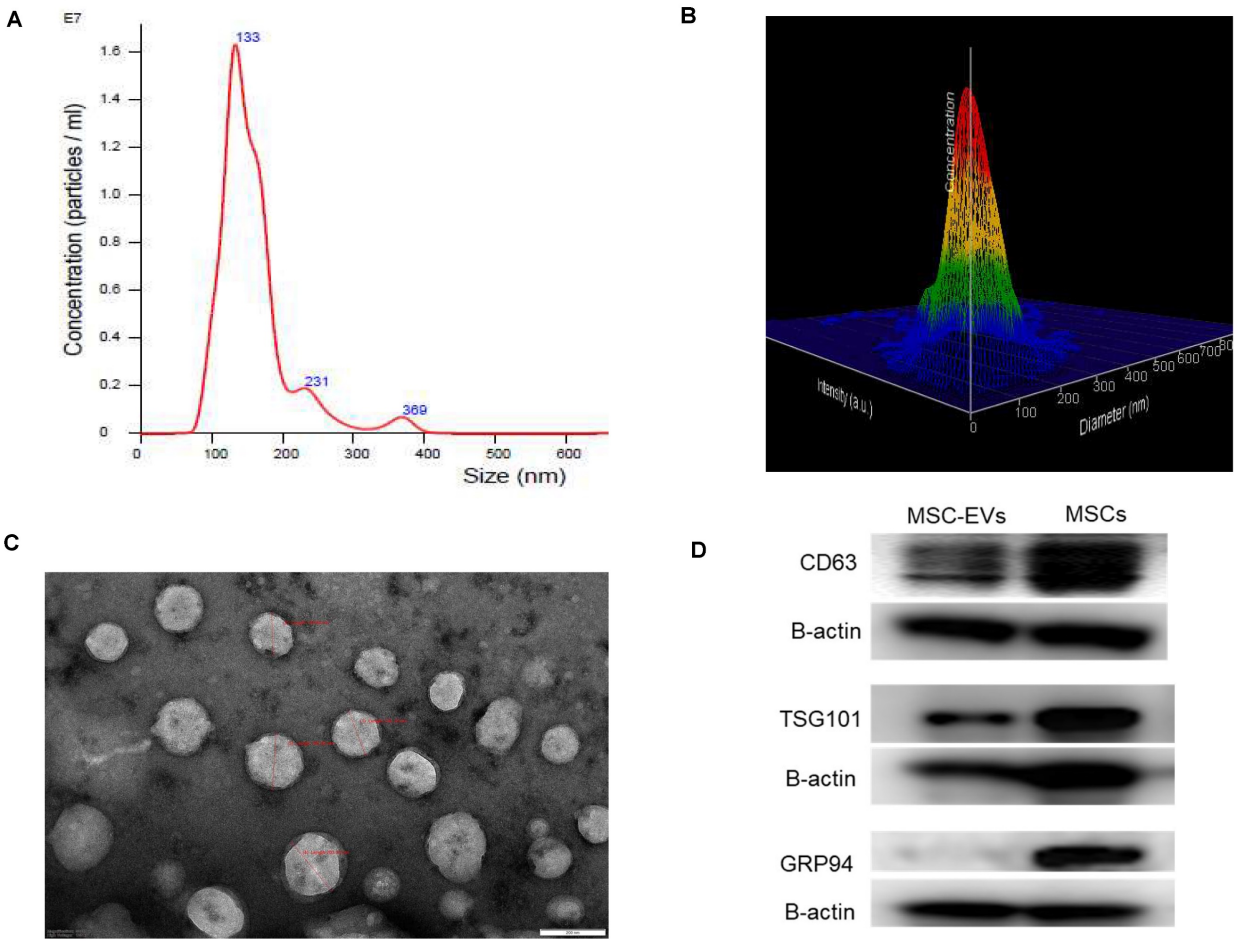
** Characterization of isolated human bone marrow derived MSC-EVs. A.** Nanoparticle tracking analysis of MSC-EVs (n = 14 independent experiments) showing the consistent presence of small extracellular vesicles with comparable concentrations. **B.** Three-dimensional view of the same picture to demonstrate the size distribution. **C.** Ultrastructural evaluation of EVs using transmission electron microscopy revealed abundant < 200 nm sized round or oval-shaped particles. **D.** Western blotting images demonstrating the presence of CD 63 and TSG 101 in the isolated sEVs and the MSCs while GRP 94 was negative in isolated EVs while present in MSCs.

Isolation of extracellular vesicles from hMSCs using the precipitation method yielded small extracellular vesicles 38 reproducibly (n = 14 independent MSC culture experiments). The mean size of the extracellular vesicles was 155.1 ± 6.3 nm (Figures 2 A and 2 B). The mode size is 117.6 ± 8.4 nm. Additional images with intensity plots were provided in Figure S2. Ultrastructural evaluation of the EVs using transmission electron microscopy revealed abundant < 200 nm-sized round or oval-shaped particles (Figure 2C). Western blotting confirmed the presence of CD 63 and TSG 101 in the isolated sEVs and the MSCs, while GRP 94 was negative in isolated sEVs while present in MSCs (Figure 2D).

### Loading efficiency and concentration of bevacizumab in sEVs

Loading of BZ to sEVs by various methods yielded nearly similar results (Figure S3). Freeze-thaw cycle, co-incubation at RT, saponin treatment and sonication resulted in loading of 61.38 ± 6.35 µg, 64.93 ± 2.86 µg, 73.63 ± 6.21 µg, 60.81 ± 5.94 µg of BZ respectively without significant difference between the methods as measured by an ELISA assay. Similarly, the freeze-thaw cycle, incubation at RT, saponin treatment and sonication resulted in the loading efficiency of 30.67 ± 3.17%, 32.43 ± 1.44%, 36.78 ± 3.09%, 30.37 ± 2.96% respectively without significant difference between the methods. BZ loading using co-incubation at RT was used for all the further studies, as it is the simplest method to perform with minimal damage to sEV membrane compared to other methods.

### EV-BZ demonstrates antiangiogenic efficacy in CAM assay

**Figure 3:**
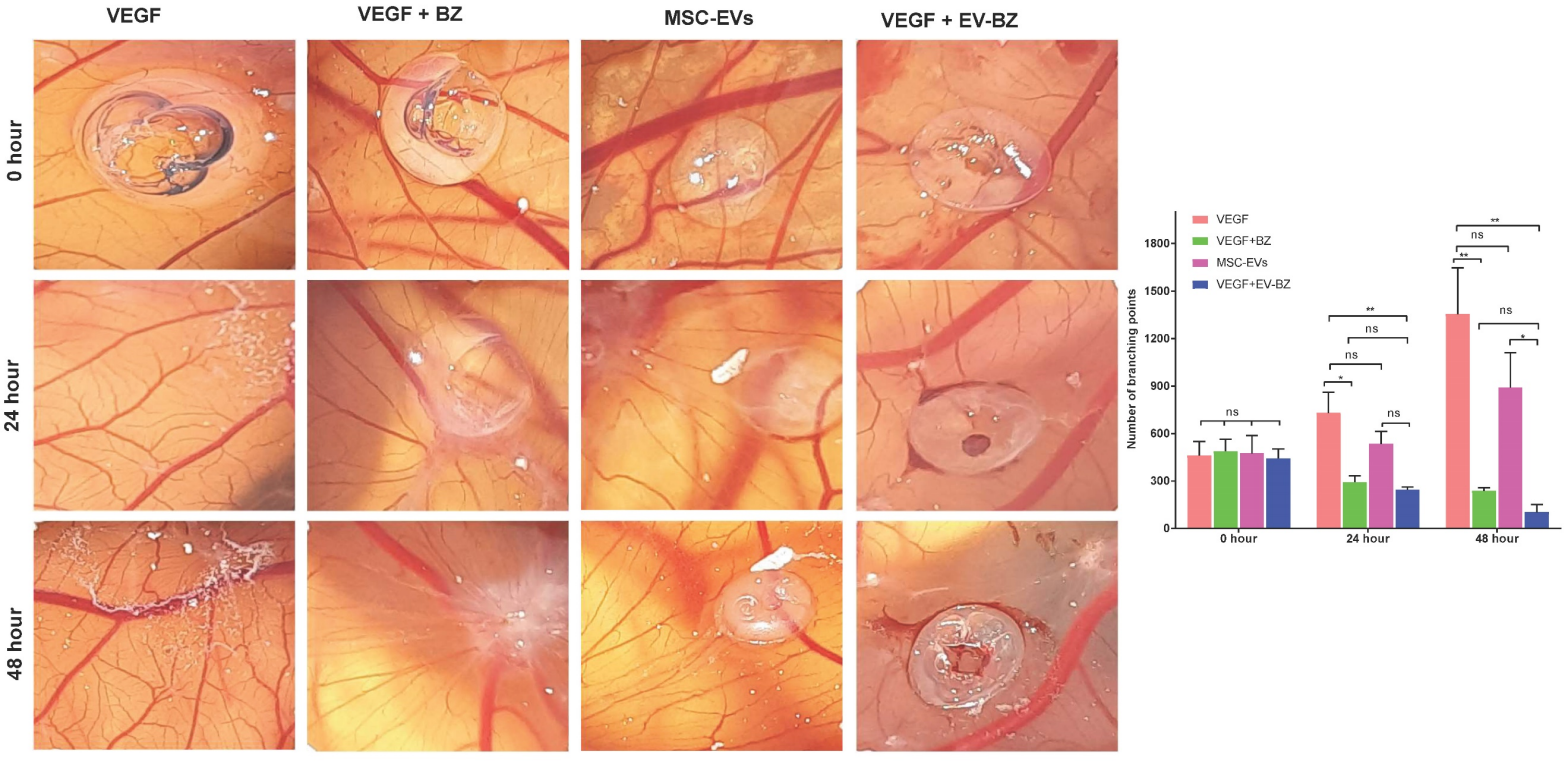
** Evaluation of anti-angiogenic efficacy using CAM assay.** The number of branching points was evaluated at 0, 24 and 48 h in the VEGF group, VEGF + BZ group, MSC-EVs group and VEGF-EV-BZ group. Values in the bar charts are presented as mean ± S.E.M for n = 5, day 9-post fertilization chicken eggs/group. **p < 0.01, *p < 0.05, ns = non-significant (One-way ANOVA).

To understand whether BZ retained its biological potency following its loading into EVs, instead of testing the efficacy in rat models directly, we have screened for the anti-angiogenic potential of EV - BZ using CAM assay (Figure 3). In a VEGF-supplemented CAM assay, the number of branching points at the start of the experiment (at 0 h) is similar in all the groups. However, at 24 and 48 h, the number of branching points was almost equally and significantly reduced in BZ (p < 0.01) and EV - BZ (p < 0.01) treatment groups. No significant difference between the MSC - EVs group and the VEGF group at 24 and 48 h while significantly reduced branching points in the EV - BZ group compared to the MSC - EV group (p < 0.05) demonstrate the partial angiogenic activity of MSC - EVs rather than antiangiogenic activity. This experiment demonstrates that BZ retained its biological potency like free bevacizumab in preventing anti-angiogenesis even after loading into EVs.

### Tracking of intravitreally injected EV - BZ in the rat retina

**Figure 4:**
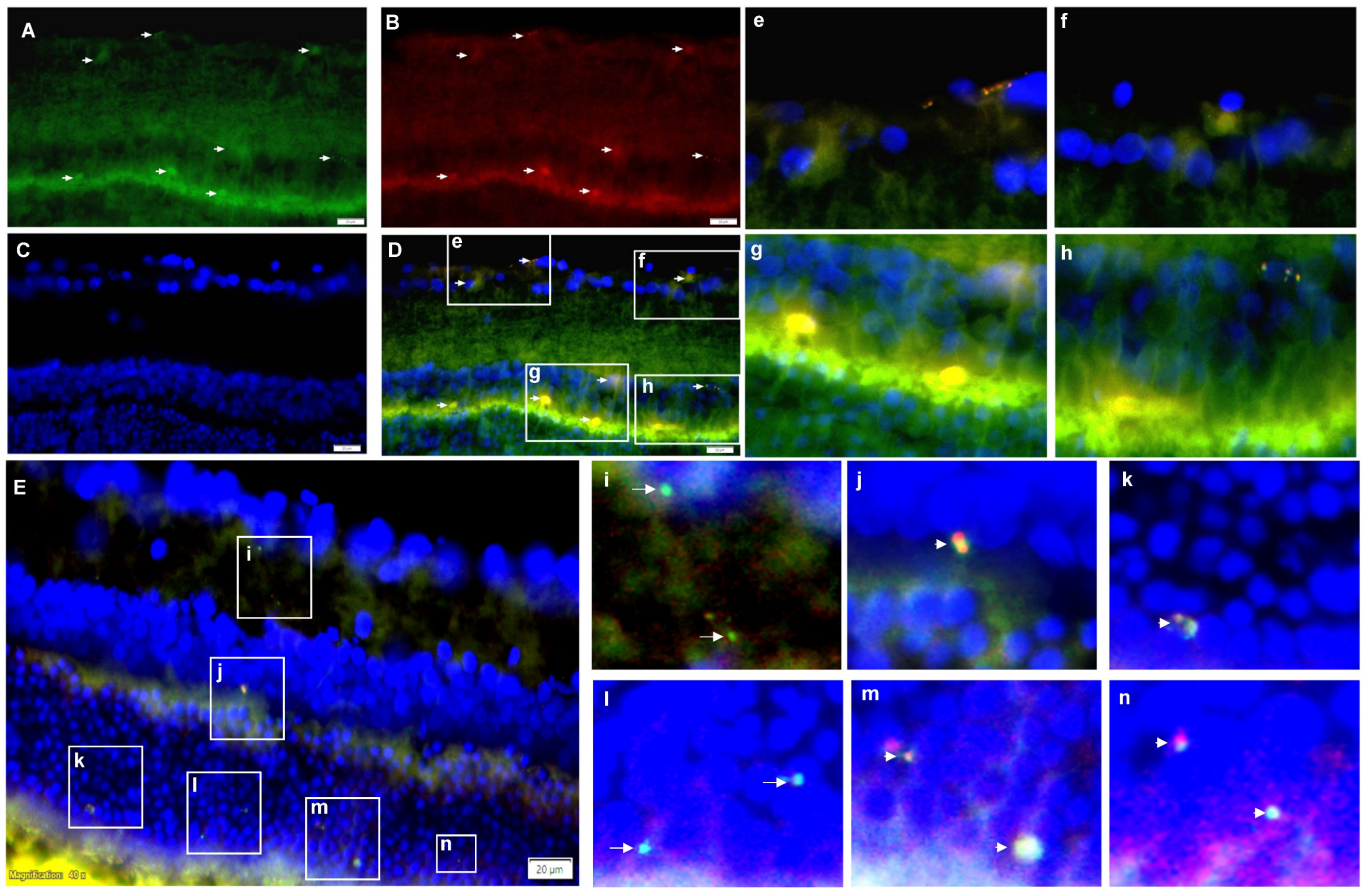
** Tracking of intravitreally injected EV-BZ in the rat retina.** For tracking EV-BZ in the rat retina following intravitreal injection, BZ was conjugated with FITC and EVs were labelled with PKH 26. We have observed FITC (green) and PKH 26 (red) labelled particles in different layers of the retina including the ganglion cell layer, inner nuclear layer, and outer nuclear layer. **A.** FITC staining. Arrows indicate FITC positive staining **B.** PKH 26 labelling. Arrows indicate PKH 26 positive staining. **C.** DAPI (blue) counterstaining. **D.** Merging **A, B and C** to locate FITC positive, PKH 26 labelled EVs in different retina layers (Orange). Selected fields from **D** were demonstrated in e, f, g and h. **E.** Demonstration of free BZ and EV-BZ in the retina through dual immunostaining for BZ and EVs. At 24 h following intravitreal injection, EV loaded with BZ (orange colour, due to red + green) and free BZ (FITC conjugated, green) could be seen in retinal layers in the extracellular matrix, suggesting BZ release from the EVs in the retina. Figures i-n demonstrate selected fields from E highlighting free BZ and EVs loaded with BZ. Arrows indicate FITC conjugated BZ, and arrowheads indicate EV loaded with BZ.

Previous studies have demonstrated that intravitreally injected MSC - EVs reached different retinal layers and remained there for several weeks 30 - 32. For tracking EV - BZ in the rat retina following intravitreal injection, BZ was conjugated with FITC (Figure S4) and EVs were labelled with PKH 26. We have observed FITC and PKH 26 labelled particles in different retinal layers, including the ganglion cell, inner nuclear, and out nuclear layers. A representative image is provided in Figures 4 A - D and 4e-4h. Also, BZ inside the EVs and free BZ in the extracellular matrix (Figure 4E and 4i-n and Figure S5), suggest BZ release from the EVs in the retina.

### EV - BZ reduces the frequency of intravitreal injection required for controlling VEGF levels in diabetic retinopathy

**Figure 5:**
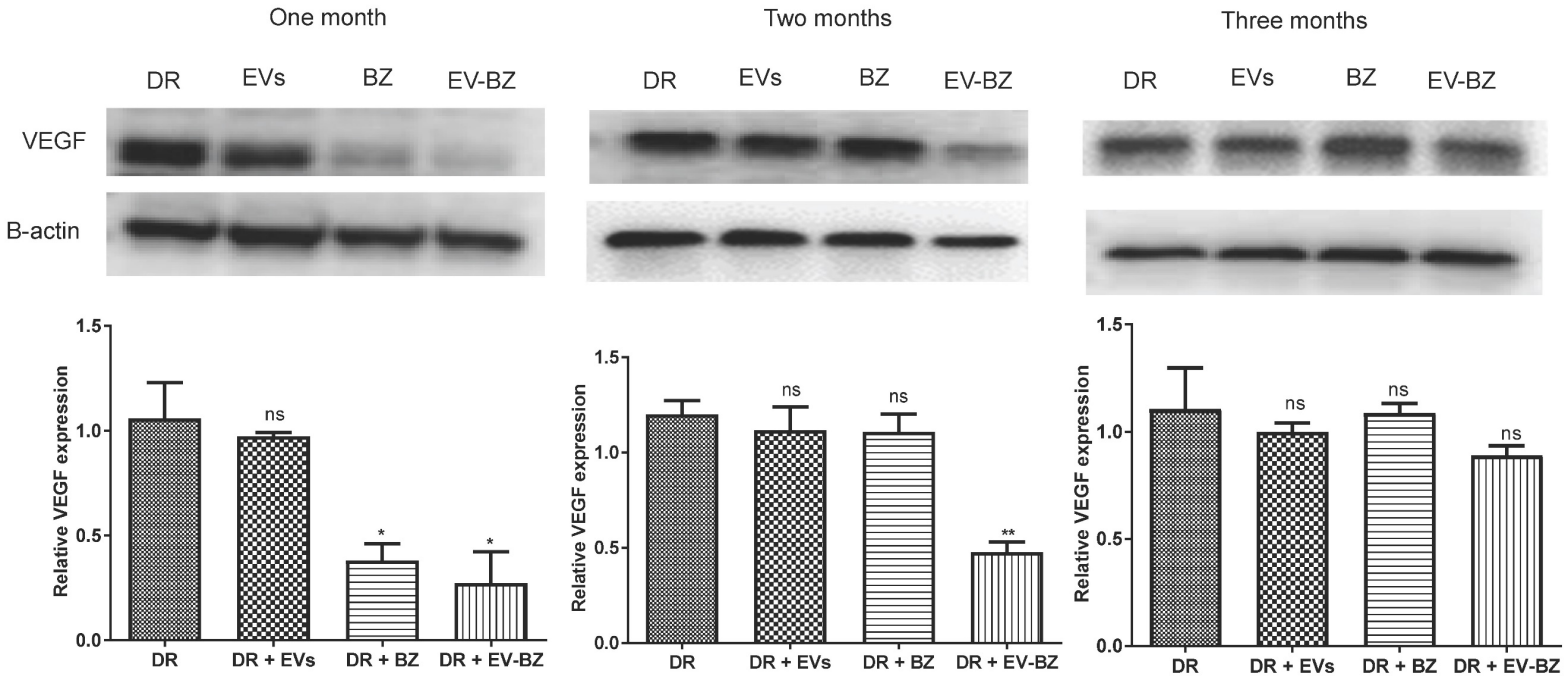
** Estimation of retinal VEGF levels in different groups.** Retinal VEGF levels were quantified using western blot at one month, two months and three months in different groups, namely diabetic retinopathy (DR) group, DR with intravitreal injection of MSC-EVs (DR + EV) group, DR with intravitreal injection of bevacizumab (DR+ BZ) group and DR with MSC-EV loaded with bevacizumab (DR + EV-BZ) group. The left panel indicates VEGF levels at one month, the middle panel indicates VEGF levels at two months, and the right panel indicates VEGF levels at three months. Values in the bar charts are presented as mean ± S.E.M for triplicate experiments. **p < 0.01, *p < 0.05, ns = non-significant (One-way ANOVA).

To understand the effect of EV - BZ in reducing VEGF levels in the retina of DR rats at 4 months of uncontrolled hyperglycemia (Figure S6), rats were intravitreally injected with MSC - EVs (n = 9), BZ (n = 9) and EV-BZ (n = 9) in different groups. Additional rats (n = 9) were used in the DR group without treatment. To quantify the VEGF levels, one set of rats was sacrificed at one month, two months, and three months following intravitreal administration (Figure 5). Analysis of the data revealed that, at one month, VEGF levels were reduced in DR + BZ group (p < 0.05) and DR + EV - BZ group (p < 0.05) compared to the DR group and DR + EV group. At the same time, no significant difference was observed between the DR group and DR + EV group. Strikingly, at two months, VEGF levels were significantly lower only in the DR + EV-BZ group compared to the DR group, DR + EV group, and DR + BZ group, while the DR + BZ group displayed similar VEGF levels compared to DR rats.

Further analysis at 3 months following intravitreal administration showed no significant difference in VEGF levels between the DR group, DR + EV group, DR + BZ group and DR + EV - BZ group. This is a novel and clinically significant finding demonstrating the sustained effects of sEV loaded with BZ (~ 2 months) over BZ (~ one month) in maintaining the lower levels of VEGF following intravitreal administration in DR to prevent angiogenesis. This finding of retinal VEGF levels at 1, 2 and 3 months suggests that sEV loaded with BZ reduces the frequency of intravitreal injection required for treating diabetic retinopathy.

To understand the available BZ levels at different time points in the retina, we have quantified, BZ using an ELISA. In the DR + BZ group, BZ levels were 13.71 ± 1.0, 10.30 ± 2.1, and 6.86 ± 1.8 ng/retina at one, two and three months following BZ administration. In the DR + EV - BZ group, BZ levels were 40.87 ± 3.3 (p < 0.01), 29.55 ± 4.4 (p < 0.05), and 14.78 ± 3.3 ng/retina (p > 0.05) at the respective time points. This suggests that BZ is preserved for a longer time when loaded with EVs. Also, it suggests that such a low difference in BZ concentration in the retina could bring significant control of VEGF levels.

### Evans blue staining demonstrated a sustained effect of EV - BZ compared to BZ in reducing retinal leakages in diabetic retinopathy

**Figure 6:**
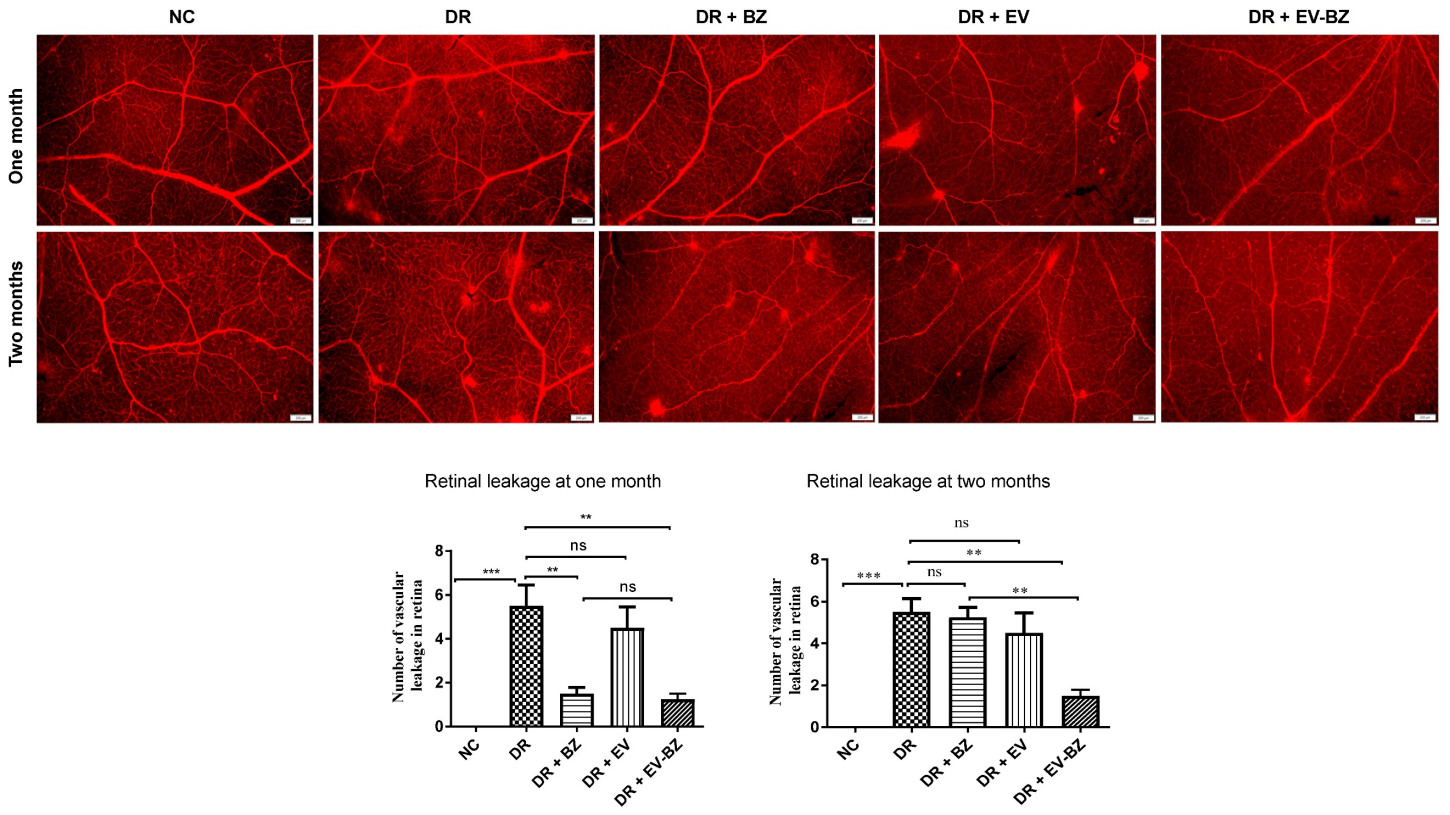
** Evaluation of retinal leakages in whole mount retina using Evans blue tracer.** Retinal leakages were quantified using Evans blue staining and evaluated in whole mount retina in the control group, diabetic retinopathy (DR) group, DR with intravitreal injection of bevacizumab (DR+ BZ) group, DR with MSC-EV group, and DR with MSC-EV loaded with bevacizumab (DR + EV-BZ) group. The top panel indicates Evans blue staining of the whole mount retina at one month and the lower panel represents Evans blue staining at two months. The table in the lower panel represents the quantified values of retinal leakages at one and two months. Values in the bar charts are presented as mean ± S.E.M for n = 5 retinas from 5 animals. ***p < 0.001, **p 0.01, *p < 0.05, ns = non-significant (One-way ANOVA).

Since VEGF levels were lower at two months following intravitreal injection of EV - BZ, we evaluated Evans Blue staining of the whole-mount retina to evaluate retinal vascular permeability at one and two months from control, DR group, DR + BZ group, DR + EV group, and DR + EV - BZ group (Figure 6). No leakages were found in the control group, while 5.5 ± 0.96 leakages (p < 0.001 versus control) were observed at one month and 5.5 ± 0.65 leakages (p < 0.001 versus control) at two months in the DR group. At one month, a significantly reduced number of leakages were observed in both DR + BZ group (1.5 ± 0.29, p < 0.01) and DR + EV - BZ group (1.25 ± 0.25, p < 0.01) compared to the DR group. However, the number of leakages in the DR + EV group was not reduced compared to the DR group (4.5 ± 0.96, p > 0.05). There was no significant difference between the control, DR + BZ, and DR + EV - BZ groups, while DR + BZ and DR + EV - BZ groups also demonstrated no significant difference.

However, at two months, the number of leakages was significantly lower only in the DR + EV - BZ group (1.5 ± 0.29, p < 0.01) compared to the DR group, while DR + BZ group demonstrated exudates similar to the DR group (5.25 ± 0.48, p > 0.05). Further, no significant difference was observed between the control and DR + EV - BZ groups. This finding supports that intravitreal injection of sEV loaded with BZ provided an extended duration of BZ effects in controlling neovascularization and retinal leakages in DR.

### Treatment with EV - BZ reduces retinal leukostasis

**Figure 7:**
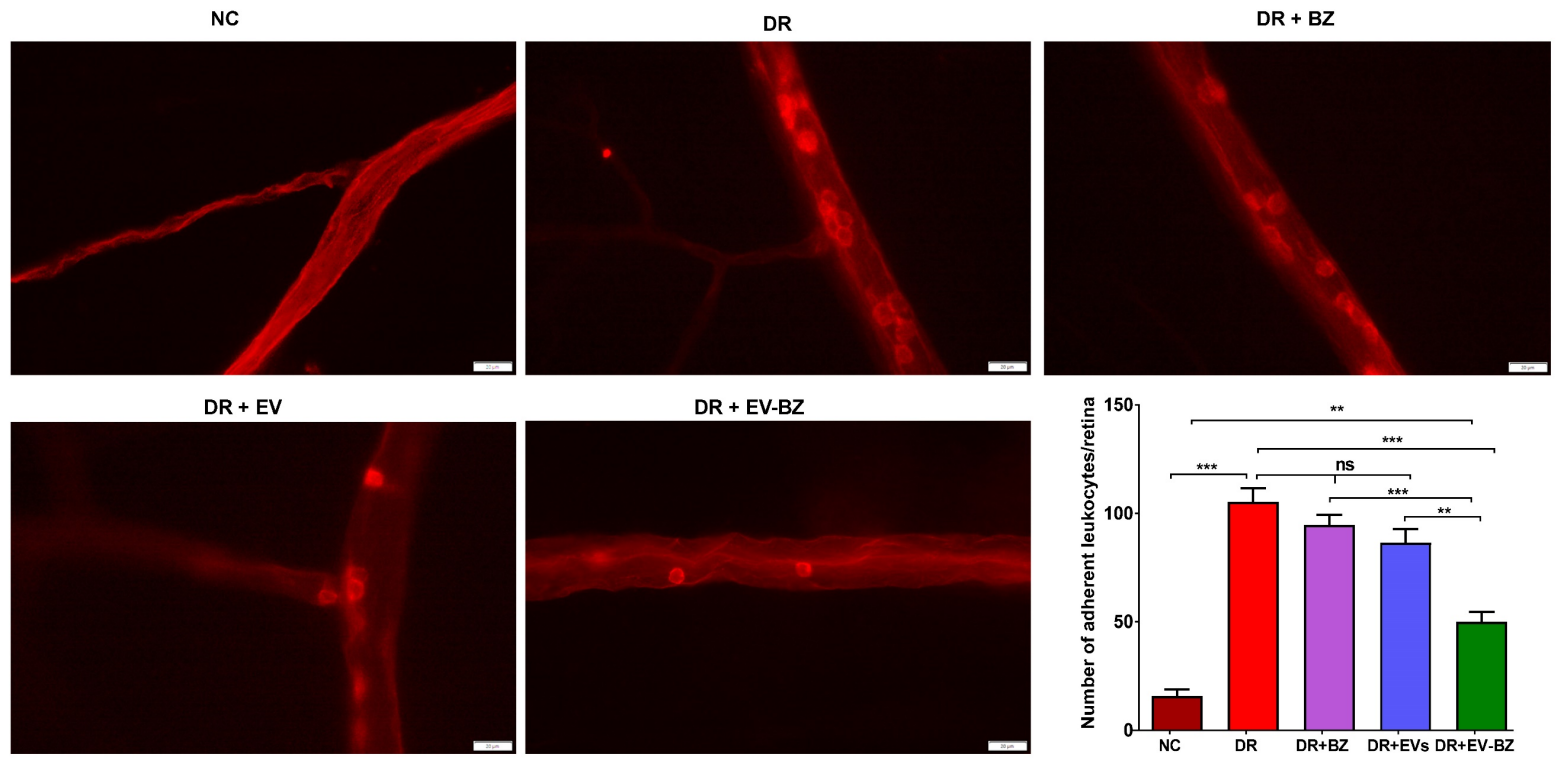
** Evaluation of retinal leukostasis using concanavalin A perfusion.** Retinal leukostasis was quantified using concanavalin A perfusion at two months following different treatments. Attached leukocytes could be seen inside the retinal vessels in the control group, diabetic retinopathy (DR) group, DR with intravitreal injection of bevacizumab (DR+ BZ) group, DR with MSC-EV group, and DR with MSC-EV loaded with bevacizumab (DR + EV-BZ) group. The table in the figure represents the quantified values of adherent leukocytes at two months in different groups. Values in the bar charts are presented as mean ± S.E.M for n = 5 retinas from 5 animals. ***p < 0.001, **p < 0.01, ns = non-significant (One-way ANOVA).

It is strongly believed that retinal vascular leakage is well associated with retinal leukostasis. The number of adherent leukocytes in the DR group is significantly high compared to the control group (p < 0.001, Figure 7). Rats with DR treated with BZ and MSC - EVs failed to reduce the number of adherent leukocytes at two months (p > 0.05). However, treatment of EV - BZ in the rats with DR demonstrated a significant reduction in the number of adherent leukocytes to the vascular surface (p < 0.001). However, the number of adherent leukocytes in the EV - BZ group is significantly higher compared to the control group (p < 0.01).

### Intravitreal injection of EV - BZ reduced cell death associated with diabetic retinopathy

**Figure 8:**
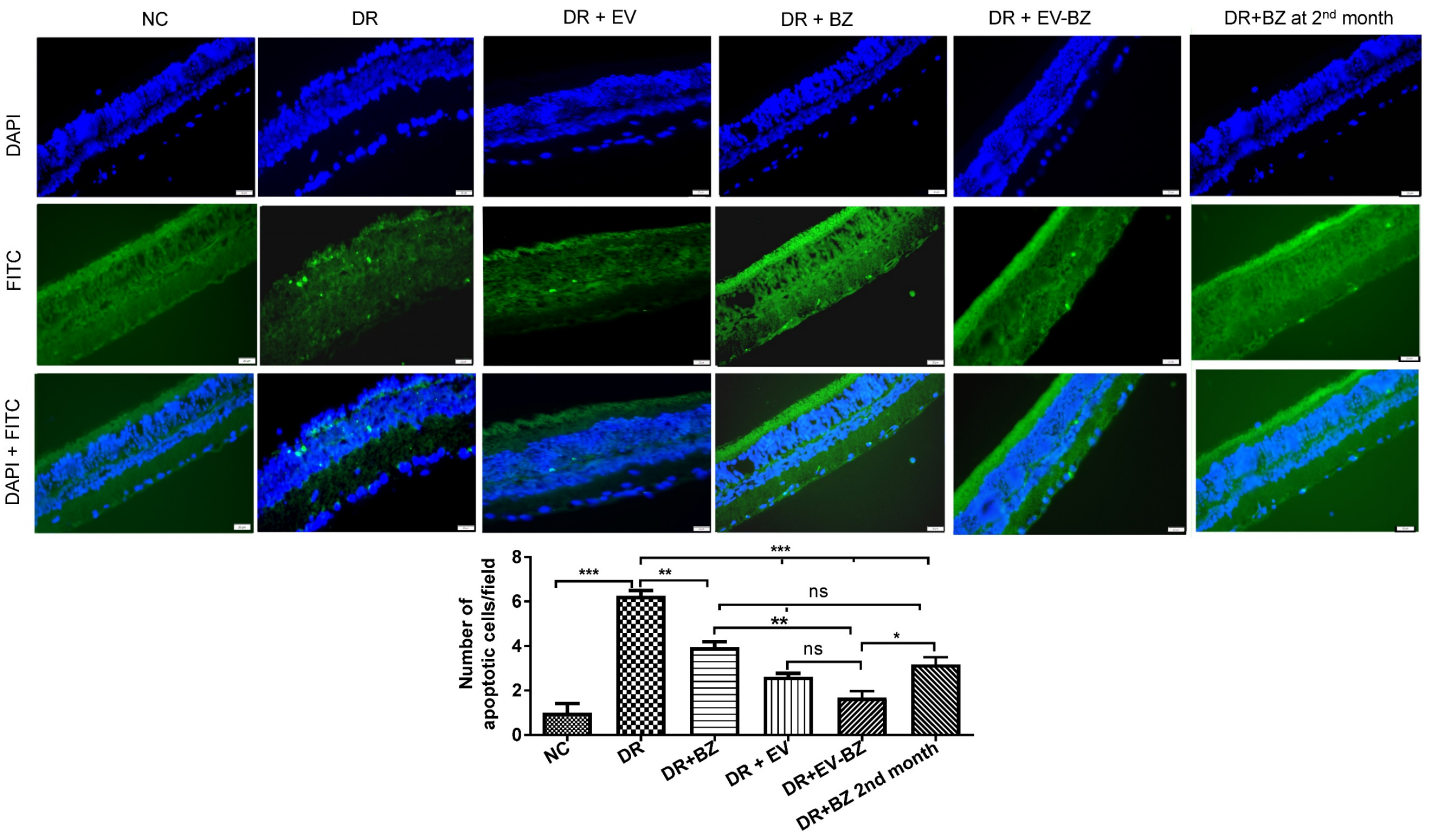
** TUNEL assay for the evaluation of the retinal cell death.** Retinal cell death was evaluated using TUNEL assay in the naïve control (NC) group, diabetic retinopathy (DR) group, DR with intravitreal injection of MSC-EVs (DR+ EV) group, DR with intravitreal injection of bevacizumab (DR+ BZ) group, DR with MSC-EV loaded with bevacizumab (DR + EV-BZ) group and an additional injection of BZ at second month (DR+BZ 2^nd^ month) group. The upper panel indicates DAPI (blue) staining, the middle panel indicates TUNEL-positive FITC (green) staining and the lower panel indicates merged images (blue + green). The table in this figure represents the number of apoptotic cells per field in different groups. Values in the bar charts are presented as mean ± S.E.M for n = 5 retinas from 5 animals. ***p < 0.001, **p < 0.01, *p < 0.05, ns = non-significant (One way ANOVA).

Multiple cell types, including the neural retina and vascular cells, undergo chronic cell death in DR, contributing to vision loss over the years. This study identified significant cell death in the DR group compared to controls (Figure 8). Evaluation of cell death at 2 months following intravitreal administration of BZ or EV - BZ demonstrated a significant reduction in cell death in DR + BZ (p < 0.01) and DR + EV - BZ (p < 0.001) groups compared to the DR group. Further, the amount of cell death in the DR + EV - BZ group is significantly low (p < 0.01) compared to DR + BZ group. Also, compared to DR, the DR + EV group demonstrated a significantly lower number of cell death (p < 0.001). However, no significant difference was observed between the DR + EV group and the DR + EV + BZ group. We have further evaluated if an additional injection of BZ in the second month has a better effect in preventing cell death than a single dose of EV - BZ. Indeed, another injection of BZ in the second month demonstrated lower levels of cell death compared to the DR group (p < 0.001) which is better than the effect observed with a single dose of BZ (p < 0.01). Interestingly, the amount of cell death observed in the DR + EV - BZ group is significantly low compared to another injection of BZ in the second month (p < 0.05). This demonstrates that EV - BZ provides additional neuroprotection against retinal cell death two months following intravitreal injection compared to monthly BZ administration.

### Intravitreal injection of EV - BZ failed to control chronic inflammation associated with diabetic retinopathy

**Figure 9:**
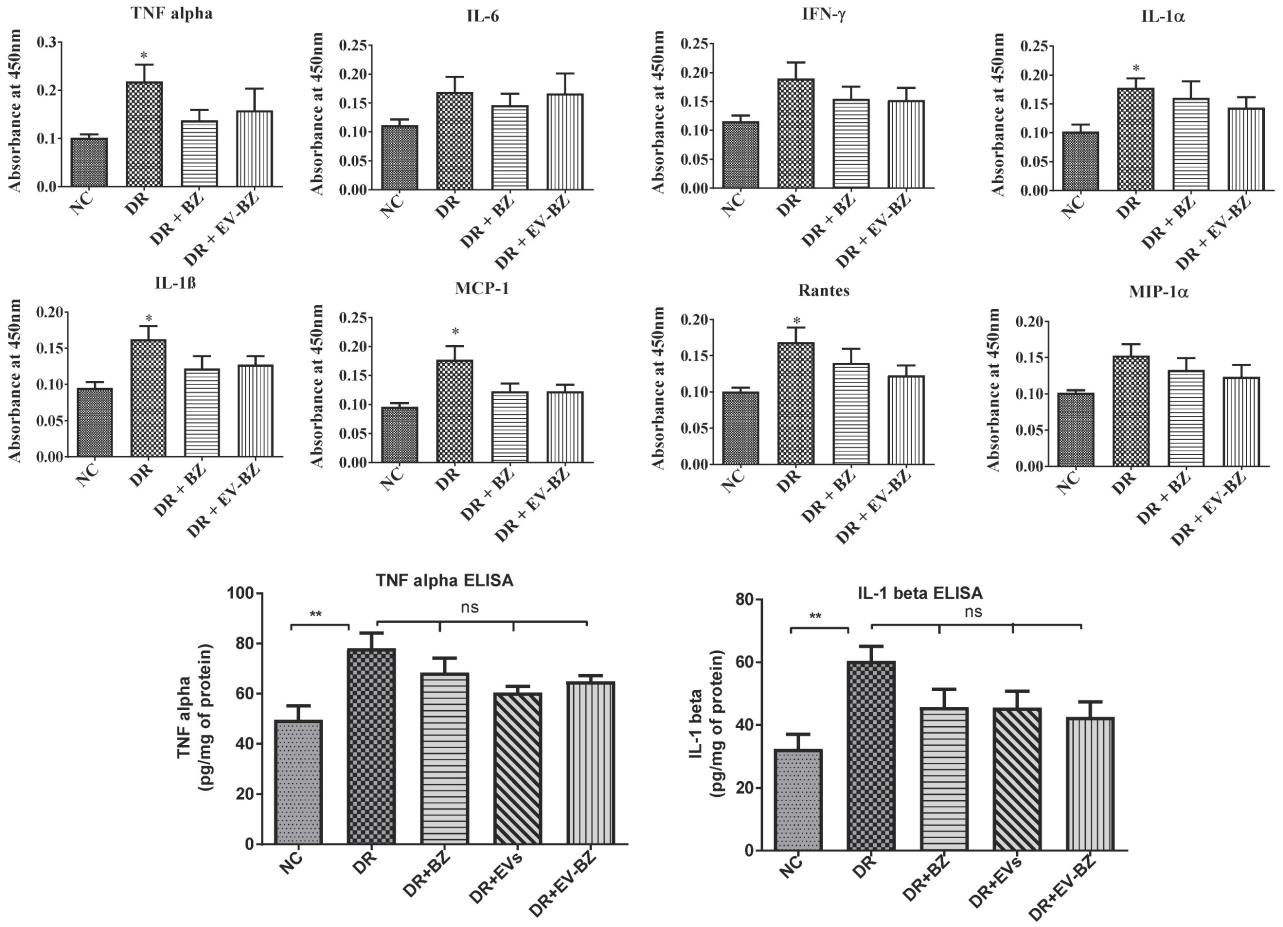
** Evaluation of retinal inflammation using ELISA for cytokines.** Chronic inflammation in the retina was evaluated using rat inflammation ELISA strips in the naïve control (NC) group, diabetic retinopathy (DR) group, DR with intravitreal injection of bevacizumab (DR+ BZ) group, and DR with MSC-EV loaded bevacizumab (DR + EV-BZ) group. The following cytokines were evaluated using the strips: TNFα, IL-6, IFN-γ, IL-1α, IL-1β, MCP-1 and rantes (upper and middle panel). In the lower panel, quantitative ELISA results were demonstrated for TNF α, and IL-1β in the control group, DR group, DR + BZ group, DR + MSC-EV group and DR + EV-BZ groups. Values in the bar charts are presented as mean ± S.E.M for n = 5 retinas from 5 animals. ***p < 0.001, **p < 0.01, *p < 0.05, ns = non-significant (One way ANOVA).

Chronic inflammation is an essential factor contriuting to the pathology of diabetic retinopathy. Chronic retinal inflammation in DR was evaluated, and the effect of treatment in controlling inflammation at two months following intravitreal administration of BZ and EV - BZ was analysed. Evaluation of multiple cytokines levels in the retina using ELISA strips reveals that TNF - α, IL - 1α, IL - 1β, MCP - 1, and rantes were significantly upregulated in the DR group compared to naïve controls (NC, Figure 9, upper two panels). However, intravitreal administration of BZ or EV - BZ failed to reduce any upregulated cytokines compared to the DR group. To confirm these findings, we have estimated retinal TNF - α and IL1 - 1β levels using quantitative ELISA (Figure 9, lower panel). This assay confirmed that TNF - α and IL1 - 1β levels were upregulated in the DR group compared to controls while intravitreal treatment of BZ, EVs, or EV - BZ to the DR rats failed to reduce their levels in the retina.

Regulating VEGF levels and reducing retinal leakages are the core of anti - VEGF treatment for DR. Reduced VEGF and retinal leakages with bevacizumab treatment are well established 9, 11-12, 21. The half - life of intravitreally injected bevacizumab in human eyes was reported to be between 2.5 to 7.3 days, with a mean value of 4.9 days 42. In animal models, the vitreous half-life of bevacizumab was demonstrated as 4.32 - 6.6 days in different studies, while very low levels of BZ were maintained at one month 43, 44. We observed a significantly higher level of BZ in the retina up to two months when intravitreal injection of EV loaded with BZ was compared to BZ alone administration. This is a possible reason for the sustained effects of EV loaded with BZ compared to BZ alone administration. To further understand the mechanism of sustained effects of sEV loaded with BZ in DR, the pharmacokinetics and pharmacodynamics of EVs in the retinal tissues need to be studied in detail. It was demonstrated that the fusion of internalized EV membranes with endosomes/lysosomes, leads to the release of cargo to the cytosolic compartment of the cell 45. Also, as secreted VEGF mostly functions in the extracellular matrix, it is possible that BZ released from the EVs into the extracellular matrix could directly control VEGF levels. As intravitreally administered sEVs are approximately one billion in this study, heterogeneous and gradual degradation over a long period could be a reason for sustained levels of BZ in the retina following EV - BZ treatment compared to BZ-only administration. Earlier studies have demonstrated that intravitreally injected MSC - EVs reached different retinal layers, and their presence was detected for several weeks in the retina 30-32. Also, as the EV - BZ group carried a lower amount of BZ than BZ alone, their sustained biological function highlights the ability of EVs to preserve the bioactivity of loaded BZ for a prolonged duration inside the tissues.

## Discussion

The present study provided significant evidence for the prolonged benefits of sEVs as a drug delivery system. Anti-VEGF therapy through repeated intravitreal injections is an established treatment for reducing VEGF levels in the retina for inhibiting neovascularization and leakage of hard exudates for preventing vision loss. Although anti-VEGF therapy has several clinical benefits, its monthly requirement potentially causes devastating ocular complications, including trauma, intraocular hemorrhage, retinal detachment, endophthalmitis, etc. A treatment that could reduce monthly intravitreal injection frequency is well considered for clinical applications. As MSCs and MSC - EVs demonstrated safety in several clinical studies, we have tested the efficacy of MSC - EV loaded anti-VEGF drug bevacizumab in controlling VEGF levels in a rat model of DR. The study identified a clinically important finding that sEV loaded with BZ reduces the frequency of intravitreal injection required for treating DR. The sustained effect is observed from the reduced levels of VEGF, the number of retinal leakages and leukostasis until two months following intravitreal injection of sEV loaded with BZ. At the same time, BZ alone could maintain reduced levels until one month. Although sEV loaded with BZ failed to reduce inflammation at 2 months, adherent leukocytes and cell death in the retina were consistently lower in this period compared to only bevacizumab.

Retinal cell death is well associated with DR 46,47. Controlling persistent retinal cell death is essential to prevent vision loss in DR. Long-term increased levels of VEGF are known to induce apoptosis. At the same time, anti - VEGF treatment is concomitant with reduced levels of VEGF and apoptosis 48. Further, the anti-apoptotic activity of therapeutic MSC - EVs in retinal pathology is well established 31, 32, 49. Lower levels of apoptotic cells in the BZ group support the earlier findings of the anti-apoptotic effect of BZ. Interestingly, a comparison of apoptosis in the EV - BZ group versus the BZ group demonstrated significantly reduced cell death in the EV - BZ group suggesting that combined treatment is superior in providing anti-apoptotic effect to retinal cells in DR. Anti-apoptotic activity of MSC - EVs is mostly attributed to their therapeutic microRNAs which could modulate cell survival pathways 50, 51.

Previous studies have demonstrated that retinal inflammation is directly linked with DR 52, 53. Increased TNF α, IL - 1α, IL - 1β, MCP - 1 and rantes indicate chronic retinal inflammation in the present study. Most of these cytokines are upregulated in diabetic retinopathy in patients 54-57. However, neither EV - BZ nor only BZ failed to reduce retinal inflammation at two months. Increased inflammation in the DR group and unaltered levels following treatment were further confirmed with quantitative ELISA for TNF α and IL - 1β, in DR + BZ, DR + EVs, and DR + EV - BZ groups. A significant number of adherent leukocytes still seen in the EV - BZ group could be a possible reason for unaltered inflammation with this treatment. Also, the present study was not targeting hyperglycemia which could be contributing to inflammatory response as hyperglycemia is directly linked with increased levels of pro-inflammatory cytokines 58 Alternatively the dose of VEGF and duration of treatment also could be contributing to the effectiveness in suppressing the inflammation as both responsiveness and unresponsiveness were reported with anti - VEGF treatments 59-60.

The anti-inflammatory activity of MSCs and derived EVs is well known 61-65. However, MSC - EVs from every individual does not possess the highest levels of anti - inflammatory potential 65. Selecting MSC - EVs with the highest anti-inflammatory potential could help to reduce inflammation following EV - BZ administration. Alternatively, sEVs loaded with a combination of anti-VEGF drugs and a nano dose of a clinically established long-acting anti-inflammatory drug could help to reduce the inflammation following intravitreal injection in diabetic retinopathy.

In summary, the study identified a clinically important finding that MSC - sEV loaded with bevacizumab reduces the frequency of intravitreal injection required for treating diabetic retinopathy. While the effect of BZ alone was observed for one month, sEV - BZ demonstrated its effects until two months as the sustained effect was observed from the reduced levels of VEGF and the number of leakages in the diabetic retina. While sEV loaded with bevacizumab provided sustained neuroprotection to retinal cells, further improvements in the methodology or additional strategies could help to curb inflammation associated with DR. Also, for retinal application, EV-mediated drug delivery systems should work as they maintain vitreous clarity in the light path due to their composition being similar to cells. This study provided significant evidence for the prolonged benefits of MSC - sEV as a drug delivery system in managing diabetic retinopathy. Also, such sustained effects could be useful for treating several types of disorders that demand repeated invasive therapeutic interventions.

## Supplementary Material

Supplementary figures.
