# Supplementary material for: Small extracellular vesicle-loaded bevacizumab reduces the frequency of intravitreal injection required for diabetic retinopathy
Source: Theranostics. 2023 Apr 9;13(7):2241–55. doi: 10.7150/thno.78426 (PMC10157735; doi:10.7150/thno.78426)
Supplement: Supplementary file 1 — Supplementary figures. [file thnov13p2241s1.pdf]

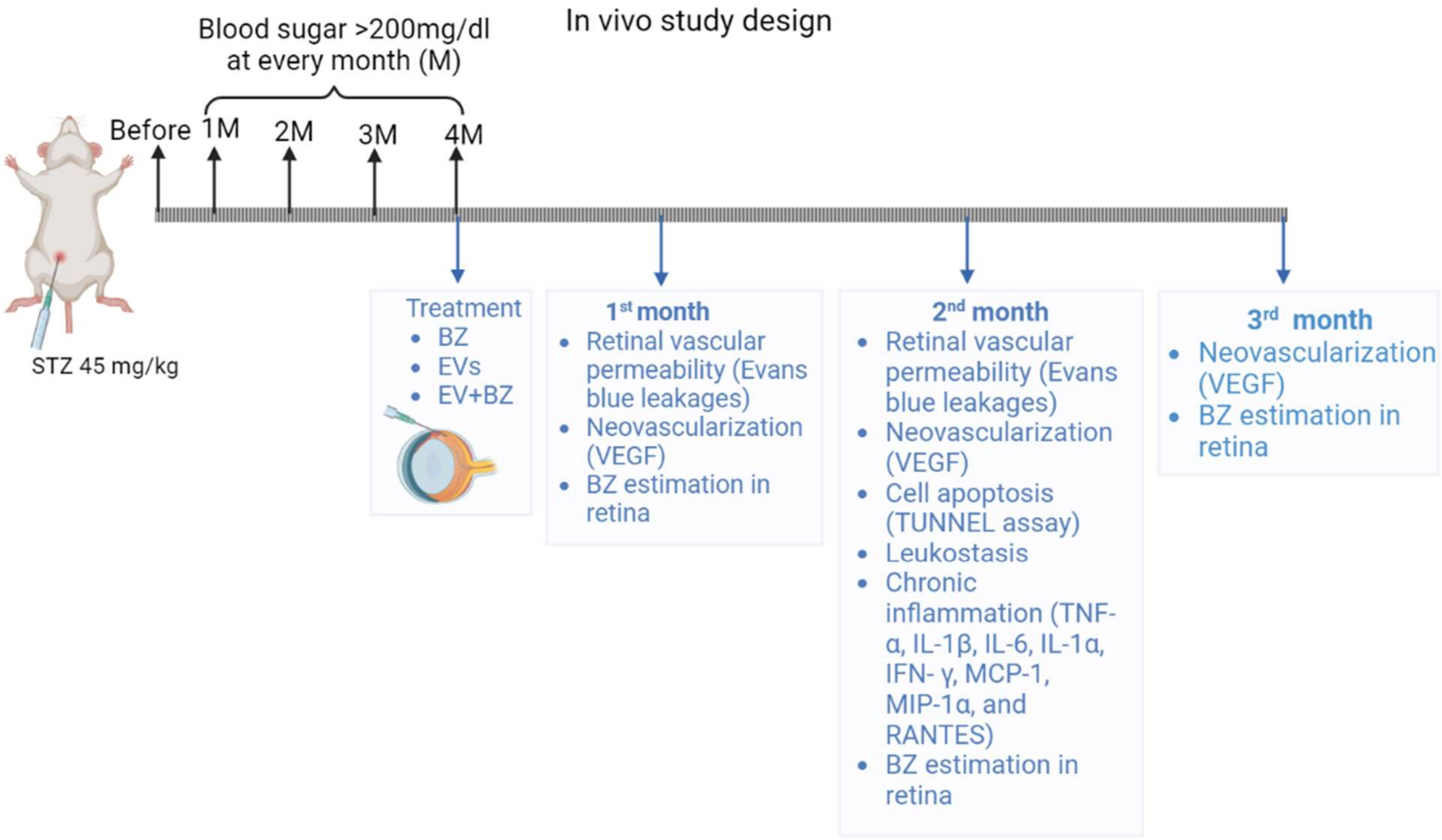

In vivo study design
Blood sugar >200mg/dl at every month (M)
Before
1M
2M
3M
4M
STZ 45 mg/kg
Treatment
• BZ
• EVs
• EV+BZ
1st month
• Retinal vascular permeability (Evans blue leakages)
• Neovascularization (VEGF)
• BZ estimation in retina
2nd month
• Retinal vascular permeability (Evans blue leakages)
• Neovascularization (VEGF)
• Cell apoptosis (TUNNEL assay)
• Leukostasis
• Chronic inflammation (TNF-α, IL-1β, IL-6, IL-1α, IFN- γ, MCP-1, MIP-1α, and RANTES)
• BZ estimation in retina
3rd month
• Neovascularization (VEGF)
• BZ estimation in retina

Figure S1: Study design for the in vivo experiments in Wistar rats

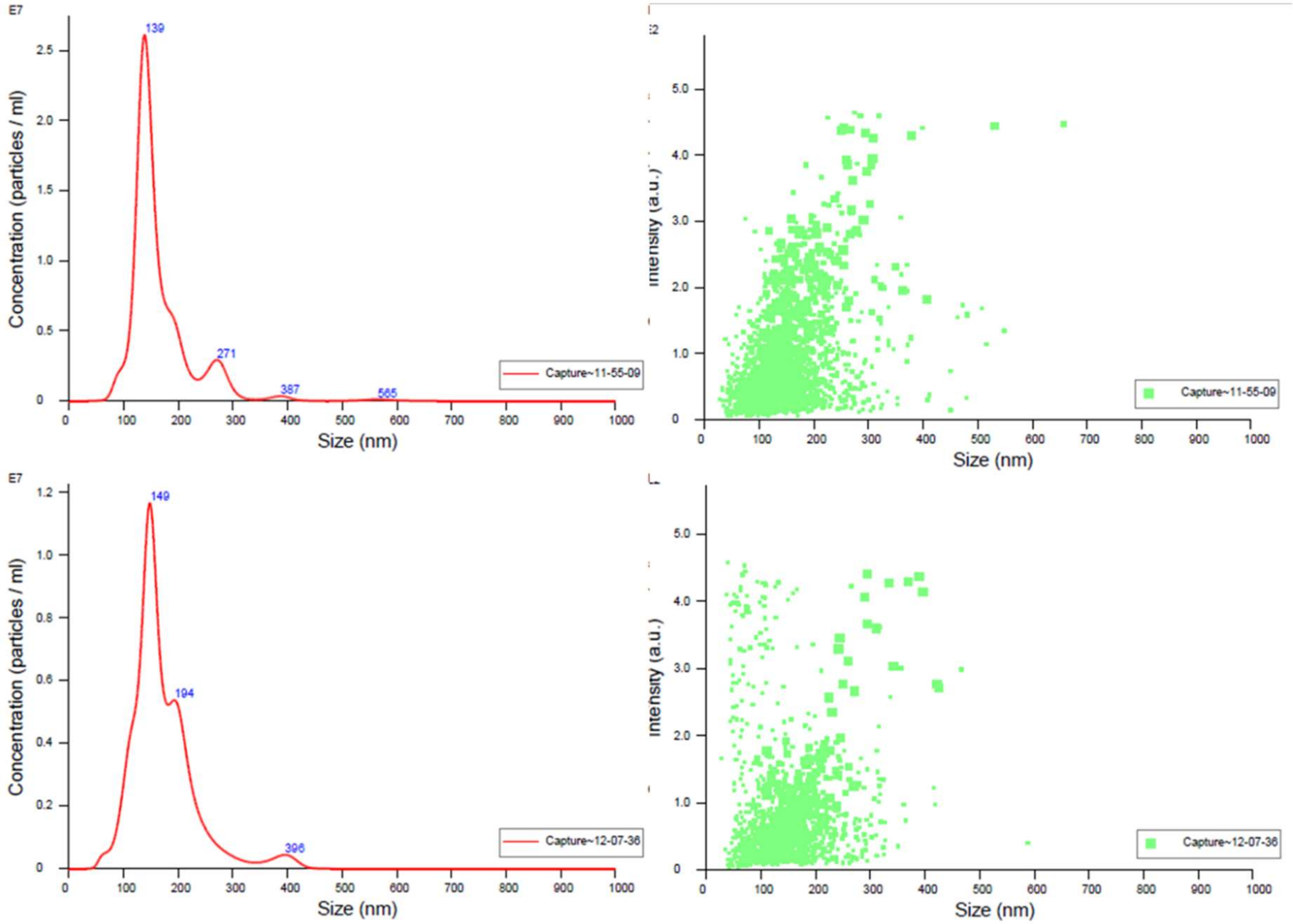
E7
Concentration (particles / ml)
139
271
387
565
Capture~11-55-09
Size (nm)
Intensity (a.u.)
Capture~11-55-09
Size (nm)
E7
Concentration (particles / ml)
149
194
396
Capture~12-07-36
Size (nm)
Intensity (a.u.)
Capture~12-07-36
Size (nm)

Figure S2: Isolated EVs are mostly small extracellular vesicles

Isolation of extracellular vesicles from hMSCs using the polyethylene glycol precipitation method yielded small extracellular vesicles reproducibly. Two additional experimental data (upper panel and lower panel) were provided with NanoSight analysis for size distribution with respective intensity in arbitrary units.

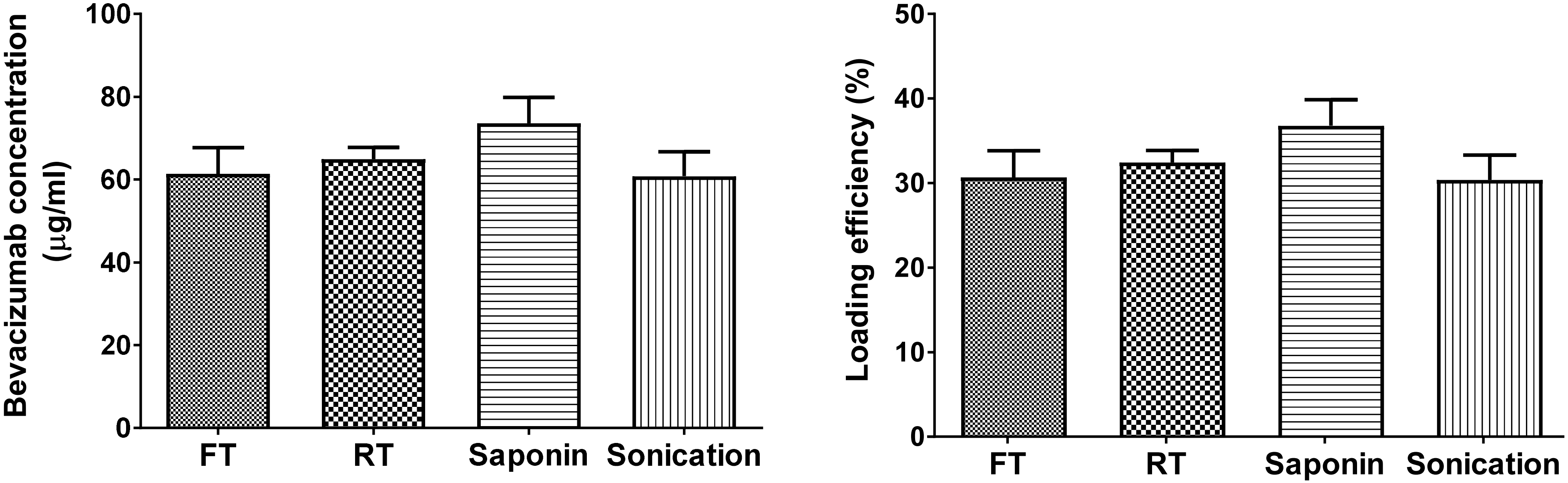
Bevacizumab concentration (μg/ml)
100
80
60
40
20
0
FT
RT
Saponin
Sonication
Loading efficiency (%)
50
40
30
20
10
0
FT
RT
Saponin
Sonication

Figure S3: Bevacizumab loading into EVs by multiple methods

Small extracellular vesicles were loaded with bevacizumab (BZ) using different methods such as freeze thaw method, co-incubation method, incubation with 0.2% saponin and sonication.

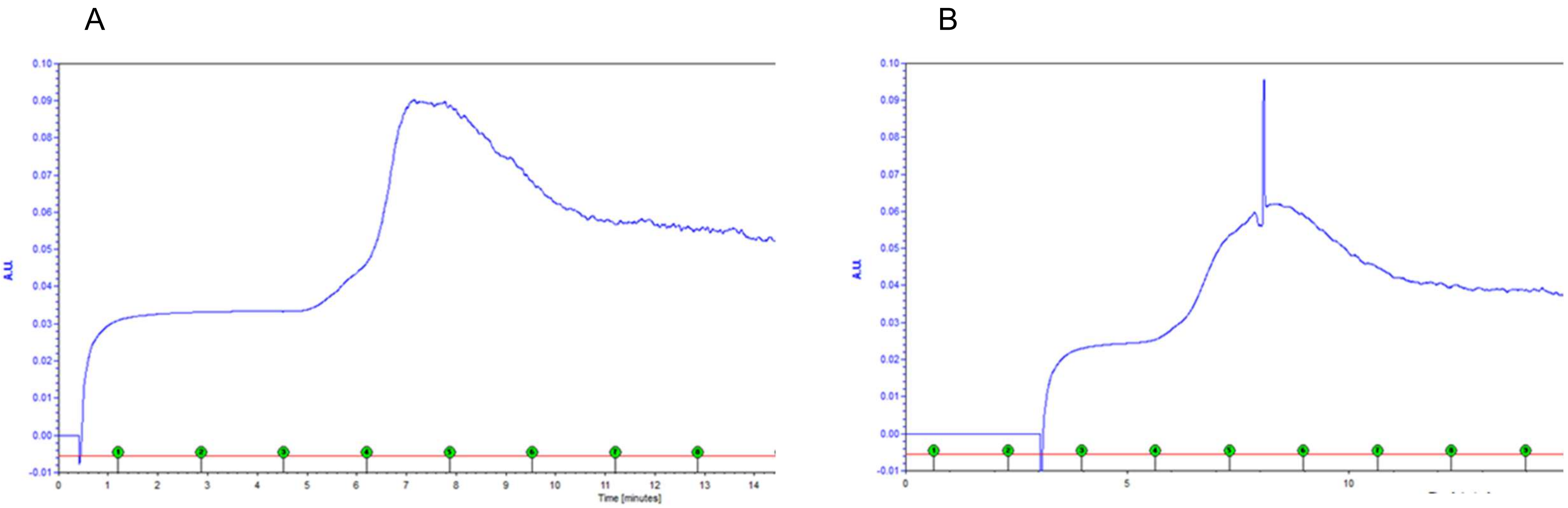
A
B
A.U.
0.10
0.09
0.08
0.07
0.06
0.05
0.04
0.03
0.02
0.01
0.00
-0.01
0
1
2
3
4
5
6
7
8
9
10
11
12
13
14
Time [minutes]
A.U.
0.10
0.09
0.08
0.07
0.06
0.05
0.04
0.03
0.02
0.01
0.00
-0.01
0
5
10

Figure S4: Conjugation of bevacizumab with FITC

A. Peak observed in figure A demonstrates bevacizumab alone. Purification of bevacizumab following derivatization started 5 min after sample injecting into column. B. Bevacizumab conjugated to FITC. Pure FITC conjugated bevacizumab started at 7 min of injecting into column.

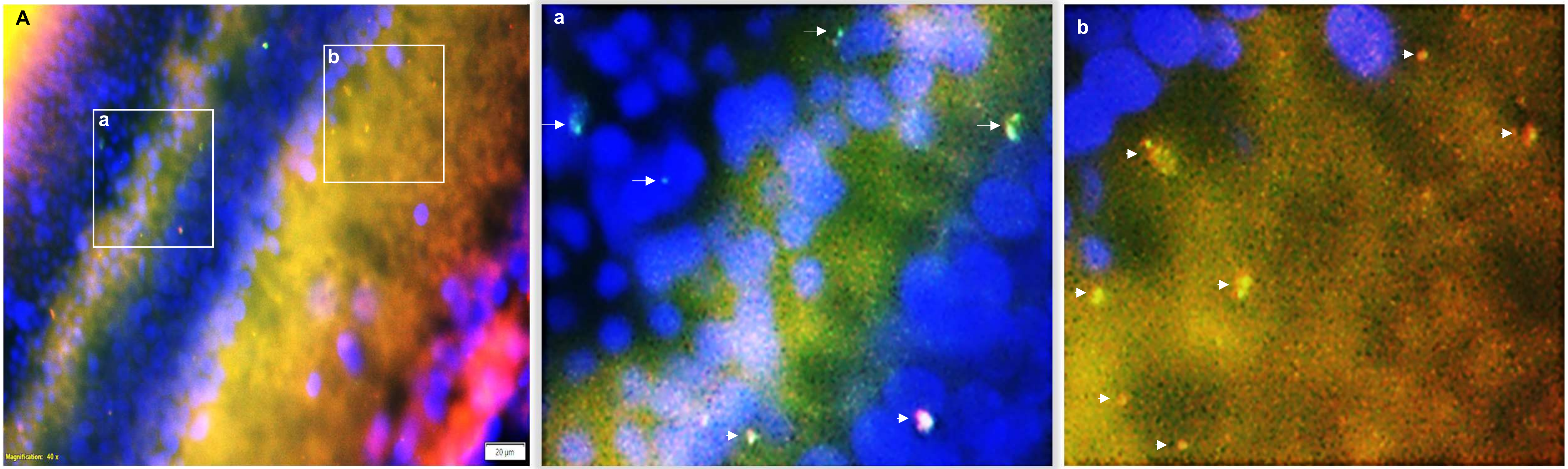
A
a
b
Magnification: 40 x
20 µm

Figure S5: Demonstration of free BZ and EV-BZ in the retina

Bevacizumab was conjugated with FITC (green) and sEVs were labelled with PKH 26 (red) before loading BZ into sEVs. At 24 h following intravitreal injection, free BZ (FITC conjugated, green) and sEV loaded with BZ (orange) could be seen in retinal layers in the extracellular matrix, suggesting BZ release from the EVs in the retina. Figure A demonstrates part of a retinal section while a and b demonstrate selected fields from A. Arrows indicate FITC conjugated BZ, and arrowheads indicate sEV loaded with BZ.

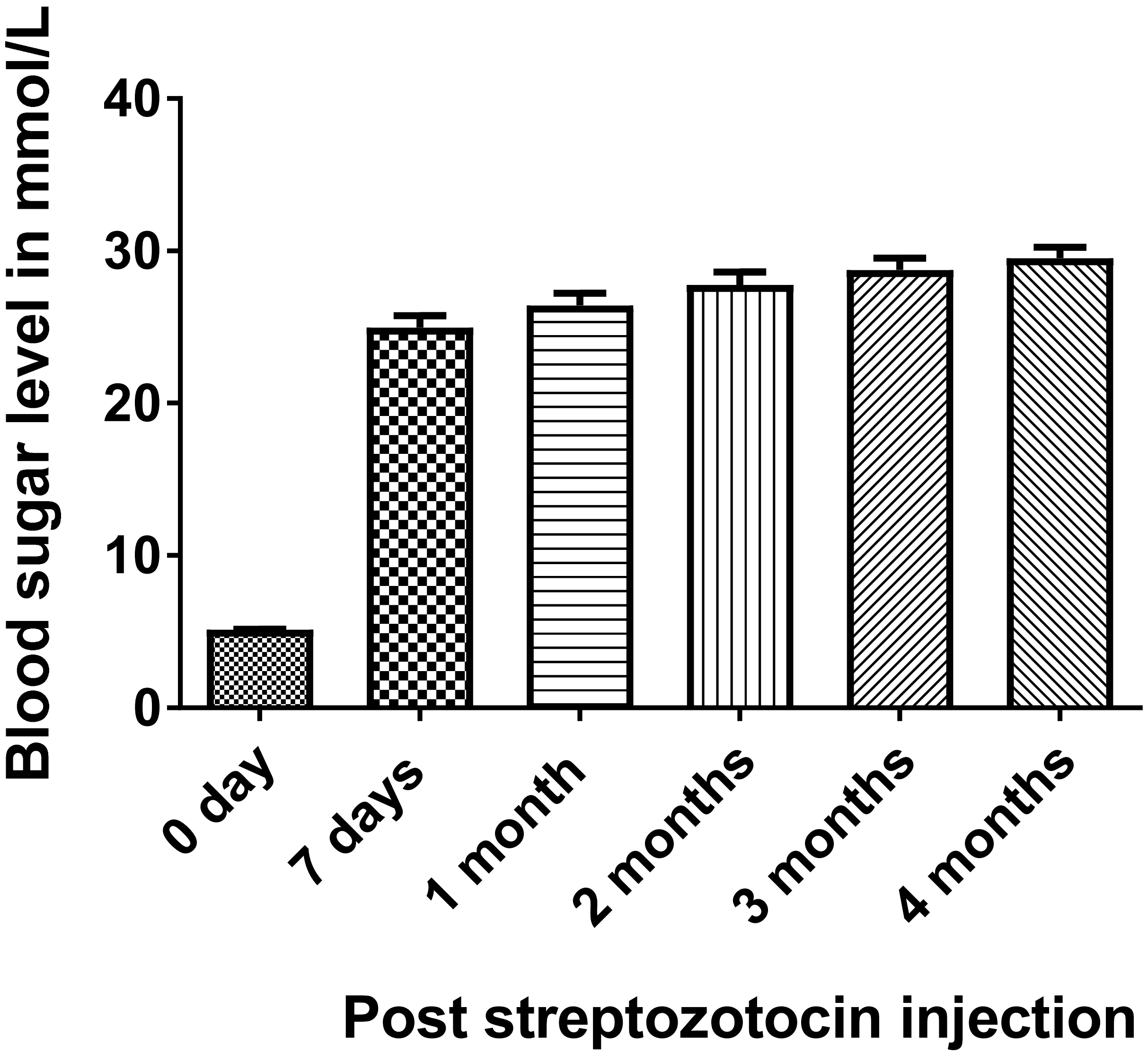
Blood sugar level in mmol/L
40
30
20
10
0
0 day
7 days
1 month
2 months
3 months
4 months
Post streptozotocin injection

Figure S6: Blood glucose levels of streptozotocin injected rats

Following streptozotocin administration, blood glucose levels were continuously monitored in rats (n = 172). Among them, 163 rats-maintained glucose levels consistently >25 mmol/L. Nine rats were excluded due to low blood glucose levels.
